# Hints to Young Practitioners—No. II

**Published:** 1872-08

**Authors:** 


### HINTS TO YOUNG PRACTITIONERS—No. II.

BY SENEX.

Emetics.—In all cases of colic, begin your treatment with a mustard emetic. Give a quart or more of fluid, if required. The patient will try to beg off, but you must not heed him. Tumbler full after tumbler full must be pressed upon him, until vomiting is produced. Never mind whether the pain is referred to the epigastric or hypogastric region—give the emetic. Neither be deterred by denials of having taken indigestible food; it may be yesterday's cabbage or surfeit. Immediately after vomiting, inject hypodermically one-third grain of morphine, and let your patient sniff chloroform. Injections and castor oil come next in order, if required. Hot mustard poultices procure some relief—not much.

In cases of flooding attending abortion, emetics are valuable. In spite of ice, the tampon and ergot, leakage will often go on, and your patient be brought to swooning, notwithstanding your

*Klebs: Pathologische Anatomie, 1869, 2 Lief, p. 276.

†Recherches Expérimentales sur la Congélation des Animaux. Journal de l' Anatomie et de Physiologie, 1866, p. 1.

brandy and water. Often a spontaneous vomiting ejects clot, fœtus, placenta, or whatever the womb contains; and from that moment, the trouble ends. You will be saved much anxiety, and many hours of vigil, and your patient be put in a condition of safety, by giving ten grains of ipecac early in the action.

In hysteria, you will find a dose of hippo very useful. The cramps and multiform antics of your patient will yield sooner to this than to valerian, Hoffman, etc. Afterward you may pursue the Abernathian philosophy, and give part of your fee to buy a skipping-rope—if you dare.

In some of the graver forms of bilious fever, you will find emetics useful. Follow a dose of ipecac with a good dose of morphia, and often there will be decisive relief. You will be compelled, out of deference to custom and the demands of friends, to give the usual doses, every two hours, of aconite, spirits of mindereri, and that sort of stuff; but you will beware of considering that you really, by such means, eliminate any of the materies morbi. Far better is the cold bath and cold pack, or sponging. Dr. Dixon recommends the douche, and Austin Flint the wet sheet. Dr. Bristone, of St. Thomas Hospital, in a recent lecture at the Royal College of Physicians, whilst deprecating the drug treatment of fever and acute diseases, extols the cold wet sheet.

In regard to alimentation in acute diseases, I think it is much overdone. I knew a physician order a sick child, to whom rest was everything, to be wakened out of sleep every hour for beef tea. It was cruel. It is all very well for Dr. Graves to have said a good thing about having it writ on his tomb that he fed fevers, but I doubt the wisdom of forcing food on a reluctant or loathing stomach. Dr. Chambers speaks of giving six or eight pints of beef tea daily. My patients would never hold so much. Better take some counsel of the instincts. Dr. Little, in a late number of the *Dublin Medical and Surgical Journal*, has some good thoughts on this subject. He likes coffee and milk, and prefers them to animal broths, particularly if there be tendency to diarrhœa. He is chary of alcohol.

As a type of a class of fevers in which emetics are useful, the following case, now under treatment, will illustrate:

August 13.—Frank Brady, aged fourteen, after usual prodrome, has high fever, loaded tongue, flushed face, injected eyes, pungent heat of skin, deafness, involuntary evacuations, partial coma. When roused, refers distress to epigastrium. Ordered twelve grains of ipecac; cold bath, to be followed by sponging; ice to the shaven scalp; hot mustard pediluvia; six grains of calomel.

August 14.—Emetic and cathartic acted well; condition the same; treatment continued. In the evening, Nature set up an epistaxis (some will have us think the doctor would surely kill by blood-letting), and in a few hours, there was manifest subsidence of fever and brain symptoms.

The case, after this, did well, requiring only a blister to the nape. The boy is still as deaf as a post, and not able to articulate a word. His diet consisted, when he could be got to swallow anything, of coffee and iced milk and beef tea.

SAVANNAH, GEORGIA, August 21st, 1872.

ERRATA.—In the first article by "Senex," published in the July number, we regret that there were two errors:

On page 233, fourth line from the bottom, for "arsenic," read *arnica*.

On page 234, fifteenth line from top, for "Armsbury," read *Armstrong*.

---

PEPSIN IN DIARRHŒA.—Dr. Davidson, of the Children's Infirmary, Liverpool, calls attention, through the *Practitioner*, to the prompt benefit which follows the use of pepsin in certain forms of diarrhœa occurring in young children. There is debility of the digestive organs; unassimilated food passes away by frequent motions, and there is often action of the bowels immediately after eating, the ingestion of food into the stomach apparently exciting the movement of the bowels. The mother states that food passes through the child as soon as it is eaten. Astringents and antacids do no good; but the writer has found that small doses of pepsin, taken three or four times a day with the food, scarcely ever fails to restore health.—*Michigan Medical Journal.*

